# Multiscale Bayesian simulations reveal functional chromatin condensation of gene loci

**DOI:** 10.1093/pnasnexus/pgae226

**Published:** 2024-06-06

**Authors:** Giovanni B Brandani, Chenyang Gu, Soundhararajan Gopi, Shoji Takada

**Affiliations:** Department of Biophysics, Graduate School of Science, Kyoto University, Kyoto 606-8502, Japan; Department of Biophysics, Graduate School of Science, Kyoto University, Kyoto 606-8502, Japan; Department of Biophysics, Graduate School of Science, Kyoto University, Kyoto 606-8502, Japan; Department of Biophysics, Graduate School of Science, Kyoto University, Kyoto 606-8502, Japan

**Keywords:** chromatin structure, nucleosomes, enhancer–promoter communication, Hi-C contact maps, molecular dynamics simulation

## Abstract

Chromatin, the complex assembly of DNA and associated proteins, plays a pivotal role in orchestrating various genomic functions. To aid our understanding of the principles underlying chromatin organization, we introduce Hi-C metainference, a Bayesian approach that integrates Hi-C contact frequencies into multiscale prior models of chromatin. This approach combines both bottom-up (the physics-based prior) and top-down (the data-driven posterior) strategies to characterize the 3D organization of a target genomic locus. We first demonstrate the capability of this method to accurately reconstruct the structural ensemble and the dynamics of a system from contact information. We then apply the approach to investigate the Sox2, Pou5f1, and Nanog loci of mouse embryonic stem cells using a bottom-up chromatin model at 1 kb resolution. We observe that the studied loci are conformationally heterogeneous and organized as crumpled globules, favoring contacts between distant enhancers and promoters. Using nucleosome-resolution simulations, we then reveal how the Nanog gene is functionally organized across the multiple scales of chromatin. At the local level, we identify diverse tetranucleosome folding motifs with a characteristic distribution along the genome, predominantly open at cis-regulatory elements and compact in between. At the larger scale, we find that enhancer–promoter contacts are driven by the transient condensation of chromatin into compact domains stabilized by extensive internucleosome interactions. Overall, this work highlights the condensed, but dynamic nature of chromatin in vivo, contributing to a deeper understanding of gene structure–function relationships.

**Significance Statement**

Eukaryotic genomes are organized into a complex of DNA and proteins called chromatin. The structure and dynamics of chromatin are intimately linked to key genomic functions such as gene regulation, but the physical mechanisms underlying such interplay remain unclear. By combining molecular models of chromatin with experimental data on mouse genome organization, we show how the 3D organization of chromatin over multiple length scales is ideally suited to support the function of several genes. At the small scale, the plasticity of chromatin allows for the formation of easily accessible conformations at gene regulatory elements, and compact ones elsewhere. At the scale of entire gene loci, the dynamic condensation of chromatin brings together distant gene regulatory elements, supporting their communication.

## Introduction

Chromatin, the complex of DNA and proteins organizing the Eukaryotic genome, plays a pivotal role in orchestrating a multitude of genomic functions, including DNA replication (1) and the precise regulation of gene activity (2). Its structure exhibits a hierarchical organization, with biological function affected by structural changes across all levels of the hierarchy (3). The fundamental unit of chromatin is the nucleosome, an assembly comprising about 150 bp of DNA wrapped around a histone octamer (4). Nucleosomes are not simply barriers preventing access to genomic DNA; rather, they can be better understood as flexible platforms on top of which key biological processes can be organized (5). For example, nucleosomal DNA sliding and unwrapping from histones modulate transcription factor (TF) binding (6–8), with downstream effects on gene expression. At a higher hierarchical level, these nucleosomes interact together to form chromatin fibers exhibiting remarkable structural polymorphism (9–11). In vivo, these fibers are predominantly disordered (12), but specific chromatin motifs are associated with distinct genomic regions, such as gene promoters or gene bodies (13). Chromatin fibers further fold into topologically associated domains (TADs), contiguous regions with enriched physical contacts separated by insulator elements (14). At the same time, phase separation drives the condensation of chromatin into large-scale compartments enriched in specific epigenetic modifications (15–18). The pattern of TADs and compartments along the genome influences the communication between cis-regulatory elements (CREs), such as enhancers and promoters (16, 19, 20). Pointing to the intimate link between genome structure and function, genetic engineering has shown a correlation between the probability of enhancer–promoter (EP) contacts and gene expression levels (21), while transcription bursting dynamics was found to be linked with EP looping over time (22).

Despite substantial progress in recent years, our understanding of the physical principles governing chromatin organization across different scales of genes remains limited, which hinders further exploration of the interplay between genomic structure and function. In this work, we integrate in vivo experimental genomic contact frequency data into polymer models to explore the 3D structure of mammalian genes from the local chromatin fiber level to long-distance interactions between CREs. Our findings shed light on how this organization can contribute to gene regulatory functions.

Theoretical approaches rooted in polymer physics have become increasingly important for complementing experiments and uncovering genomic structure–function relationships (23). Some of these approaches are data driven (24–29), with interactions between genomic sites optimized to match the experimental Hi-C contact frequencies (15). Others are centered around a key mechanism responsible for 3D genome organization, such as cohesin loop extrusion (30), bridging mediated by proteins (31), or phase separation dependent on specific epigenetic modifications (18, 32, 33). Furthermore, advances in the development of bottom-up molecular models of chromatin (34–37), parametrized based on the physics of its biomolecular components, now offer a viable route to provide a mechanistic understanding of genes based on first principles (38). Such models have revealed some of the molecular determinants of chromatin organization, including the role of nucleosome plasticity in mediating liquid–liquid phase separation (39). Notably, Bascom et al. utilized a nucleosome-resolution model to explore the structure of the HOXC gene locus in mouse embryonic stem cells (mESCs) (40), identifying the spontaneous formation of loops linking gene promoters with other regions of the locus. However, such physics-based chromatin models typically do not capture the effects of all the factors constituting the complex cellular environment, such as potential effects originating from TF binding (31, 41). Due to the practical consideration in modeling the behavior of several contributing molecular factors, physics-based models may not always accurately reproduce the experimental organization of chromatin in vivo, as determined from Hi-C or Micro-C contact frequencies (15, 42).

To address this limitation, Bayesian methods offer a framework to incorporate experimental observations into an existing physical model (43, 44). Metainference is one such Bayesian approach, originally developed to integrate NMR data into all-atom force fields for protein molecular dynamics (MD) simulations (44). We introduce and validate Hi-C metainference, an extension of the original approach to augment an existing prior model of chromatin to account for the observed in vivo contact frequencies of a target locus (from Hi-C (15), Micro-C (42), or other related approaches (13)), allowing one to characterize its 3D organization by MD simulations. As priors, we employ two distinct bottom-up coarse-grained models of chromatin: the 1CPN model (35) at nucleosome resolution, previously parametrized from near-atomistic nucleosome simulations (45), and a chromatin polymer model at 1 kb resolution newly parametrized from extensive 1CPN simulations. Therefore, our exploration of the 3D genome organization can be regarded as a two-way process where the bottom-up prior is adjusted in a top-down fashion by integrating the experimental contact frequencies, retaining the advantages of both physics-based and data-driven approaches.

In this study, we integrate Micro-C data (46) into our chromatin models and investigate the organization of the Sox2, Pou5f1, and Nanog genomic loci in mESCs. First, our simulations show that in vivo genes adopt relatively compact conformations, with the same size scaling of a crumpled globule (47), while at the same time, displaying significant structural heterogeneity and fast dynamics. Using the nucleosome-resolution prior chromatin model for a section of the Nanog locus, we find that the local organization of the chromatin fiber is highly dependent on the underlying epigenetic marks, being open at CREs and more compact elsewhere. Finally, we demonstrate that transient, direct contacts between the Nanog promoter and its nearest enhancer are driven by the condensation of the chromatin fiber into a compact domain through extensive nucleosome–nucleosome interactions. Our simulations suggest that chromatin plasticity over multiple length scales provides a physical basis for essential gene regulatory functions.

## Results

### Bayesian integration of Hi-C data into prior physics-based chromatin models

**Fig. 1.**
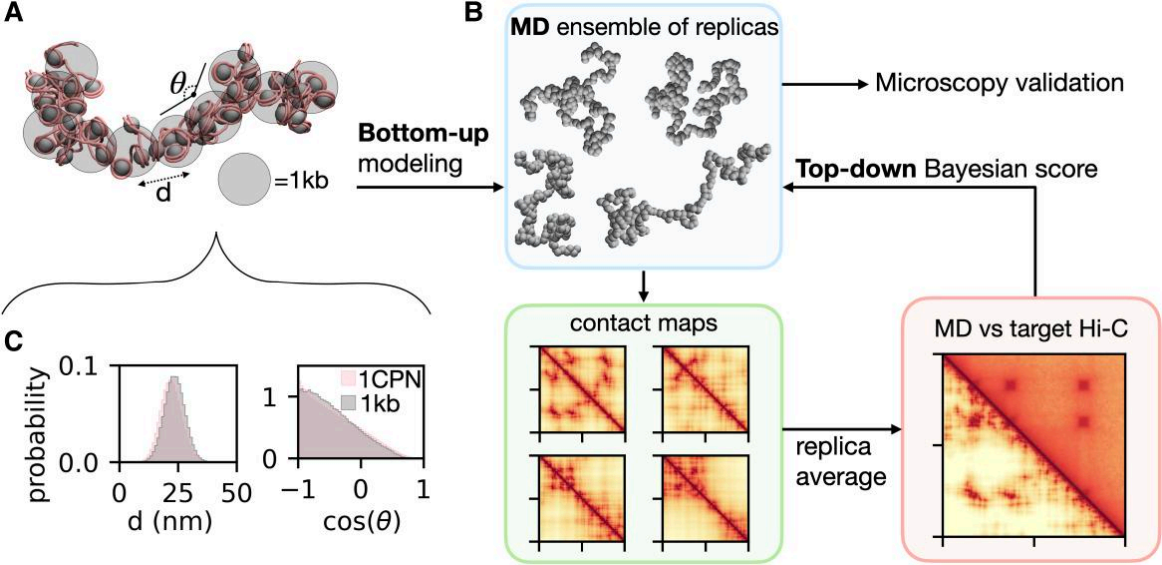
Schematics of Hi-C metainference approach. A) Representations of the nucleosome-resolution 1CPN and 1 kb prior chromatin models, indicating the 1 kb model bond distances (*d*) and angles (*θ*) whose potentials are parametrized based on the 1CPN simulations. In the 1CPN structure, nucleosomal DNA is represented as a tube, while the histone octamers are represented as spheres. In the 1 kb model, each coarse-grained bead represents five nucleosomes. B) In Hi-C metainference, we first simulate a target system using a conformational ensemble of *N* replicas based on the prior bottom-up model (e.g. *N* = 4, top panel, for a polymer of 200 1 kb beads). For each conformation, we generate a contact map using a forward model (bottom left). We average the contact maps over the replicas to generate a map of contact probabilities to be compared with the Hi-C data (bottom right). A top-down Bayesian energy score is added to the original interaction potential of the prior model to integrate the Hi-C data into the simulation. The conformational ensemble is refined over the course of the hybrid bottom-up/top-down MD simulation, and it can then be validated against independent experimental data (for example, based on microscopy). C) Illustration of the bottom-up modeling approach showing the comparison of the probability distributions of the bond distances (left) and angles (right) between 1 kb coarse-grained particles from the 1 kb resolution and the 1CPN chromatin models.

We introduce Hi-C metainference to update an existing prior model of chromatin (Fig. 1A, e.g. nucleosome-resolution 1CPN (35) or coarser 1 kb resolution model) using in vivo genomic contact frequencies from experiments, such as Hi-C (15) and Micro-C (42). Within this framework, MD simulations are used to sample the conformational ensemble of the target system from the posterior probability distribution according to the Bayes’ theorem (44). The infinite ensemble is represented using a finite number *N* of replicas of the same chromatin system—for example, a polymer made of L beads, each representing a certain number of base pairs of genomic DNA (Fig. 1B, top left panel, for *N* = 4 conformations of an *L* = 200 polymer made of 1 kb beads). The use of replicas makes metainference ideally suited to model ensemble-averaged experimental data produced by highly heterogeneous systems, such as chromatin (48). Given a chromatin structure, we use a forward model that computes the *L* × *L* contact map of each replica (Fig. 1B, bottom left panel). We then average the contact maps over the *N* replicas to compute a map of contact probabilities, which can be compared with the contact frequencies measured in experiments (Fig. 1B, bottom right panel). The dynamics of the *N* replicas is then simulated in parallel using the interaction potential of the prior chromatin model together with an additional Bayesian energy score accounting for the mismatch between experiments and predictions from the MD ensemble. This bias introduced by the Bayesian inference can be regarded as representing in vivo effects originating from epigenetics (25, 32, 49), TF binding (31, 41), and cohesin loop extrusion (30), which are not explicitly included into the physics-based model. These steps are repeated each MD timestep until we obtain a converged conformational ensemble (see Materials and methods for more details). The final ensemble may be further validated by independent experiments probing chromatin ensemble properties, for instance through microscopy (50).

In principle, Hi-C metainference can be applied using any prior MD model of chromatin, as different models cater to the needs of different applications. In this work, we use two chromatin prior models at different resolution: the nucleosome-resolution 1CPN chromatin model (35), and a coarse-grained 1 kb resolution model parametrized from 1CPN. The 1CPN model has been rigorously parametrized using a bottom-up procedure based on simulations of nucleosome–nucleosome interactions at near-atomic resolution (45), and validated against experimental chromatin sedimentation coefficients. In the 1 kb model, chromatin is represented as a polymer consisting of 1 kb beads (=5 nucleosomes) connected by bond and angle potentials (Fig. 1A, see Materials and methods for more details). The interaction parameters are optimized in a bottom-up way to match the distributions of bond distances and angles from long 1CPN simulations of 50-nucleosome fibers with a nucleosome repeat length of 200 bp (Figs. 1C and S1).

### Validation by copolymer model

**Fig. 2.**
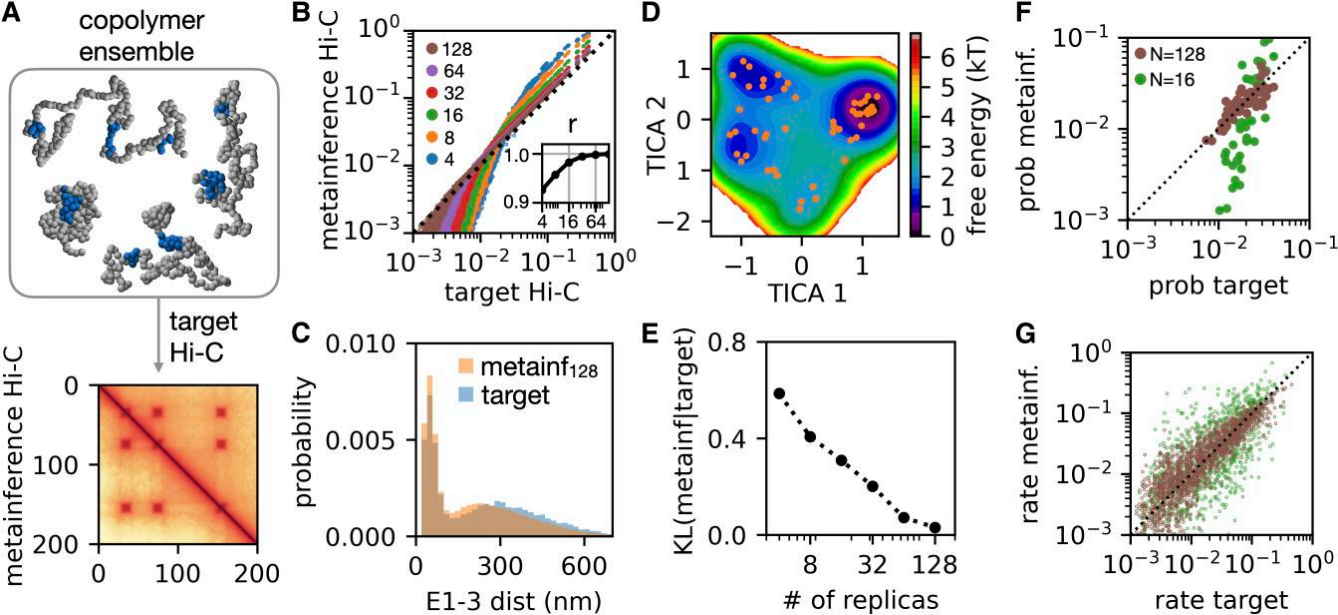
Hi-C metainference validation on complex copolymer ensemble. A) Target Hi-C map (right upper half in the bottom plot) generated by simulations of a 200-kb copolymer containing three sticky CREs, vs. the Hi-C map generated from metainference (left lower half) simulations using *N* = 128 replicas and the prior 1 kb chromatin model. B) The comparison between the Hi-C contact probabilities computed from metainference MD (*y*-axis) and from the original copolymer ensemble (*x*-axis), for a different numbers of replicas. The inset shows the Pearson correlation coefficient (*r*) between the metainference and target contact probabilities as a function of number of replicas. C) Comparison of the probability distributions of the distances between the CREs E1 and E3 obtained from the target copolymer ensemble and metainference MD with *N* = 128 replicas. D) Free energy landscape of the *N* = 128 metainference simulations projected on the first two TICA coordinates, showing the locations of the 50 cluster centers. E) Kullback–Leibler (KL) divergence of the metainference cluster probability distribution from the target copolymer distribution, as a function of the number of replicas. F and G) Comparison of the cluster equilibrium probabilities (F) and Markov state model–estimated transition rates (G) between the metainference MD (*y*-axis) and the target copolymer system (*x*-axis) for *N* = 16 and *N* = 128 replicas.

In order to test the capability of metainference to reconstruct a target polymer ensemble from its Hi-C map, we setup a test using a 200-kb copolymer made of (*L* = 200) 1 kb beads modeled as the prior chromatin model, except that three 10 kb regions (E1, E2, and E3) act as sticky CREs with relatively strong interactions with each other and weaker interactions with the rest of the locus (gene bodies like). This model produces a complex copolymer ensemble with a variety of interactions between the CREs, where these can be paired in the three possible combinations, or all be far away from each other, or all collapse into one single hub (Fig. 2A, top panel). We tested whether metainference using the noninformative prior chromatin model and the copolymer contact map can reconstruct both the statistics and the dynamics of the target copolymer.

Figure 2A shows the good agreement between the target contact map and the one generated from the metainference simulations using *N* = 128 replicas. The correlation between the contact probabilities increases, as the number of replicas increases from 4 to 128 (Fig. 2B). This is to be expected, since reproducing the formation of a contact with probability *P* requires roughly *N* = 1/*P* or more replicas, as the contact should appear at least once in the ensemble of replicas used to represent the true infinite ensemble during the MD simulation. Hi-C metainference can also recover properties not directly used as a bias, such as the distribution of distances between the different CREs (Figs. 2C, S2A and B) and radius of gyration (Fig. S2C). To provide a rigorous comparison of the ensemble statistics, we divided the conformations observed in the copolymer and metainference simulations into 50 clusters using *k*-means (51) after performing a time-structure based independent component analysis (TICA) dimensionality reduction (52) (see Supporting Information [SI] Methods). First, the free energy landscape of the *N* = 128 metainference system projected on the first two TICA coordinates (Fig. 2D) is close to the one obtained from the target copolymer simulations (Fig. S2D). The equilibrium probabilities of the different clusters are in good agreement (Fig. 2F), and the agreement, as measured by the Kullback–Leibler divergence between the distributions, increases with the number of replicas used (Fig. 2E). Finally, we confirmed that metainference can also recover the dynamics of the target copolymer system, as shown by the good correlation between the cluster transition probabilities estimated by Markov state modeling (53) of the metainference and target copolymer simulations (Fig. 2G, see SI for details of the Markov state modeling).

### mESC chromatin has a compact but heterogeneous 3D organization

**Fig. 3.**
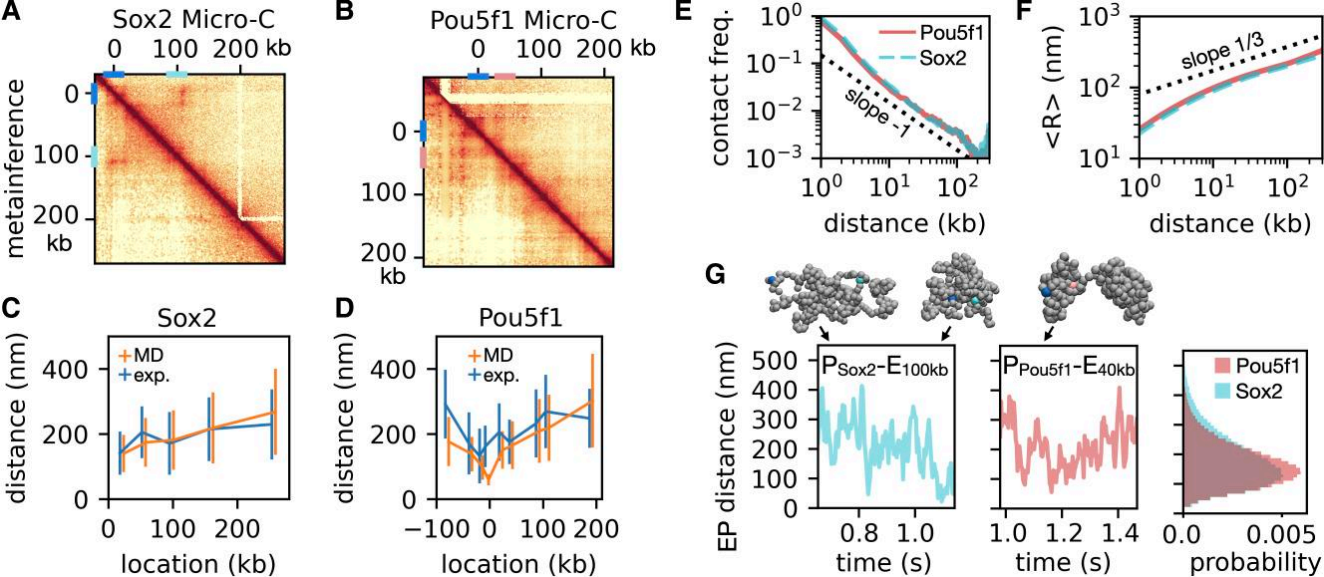
Structural heterogeneity of the Pou5f1 and Soc2 loci of mESCs. A and B) Comparison of Micro-C contact maps of the 300 kb loci of the Pou5f1 (A) and Sox2 (B) genes with the corresponding results obtained from metainference with *N* = 128 replicas. The genomic locations of the two promoters (0 kb), the Sox2 + 100 kb enhancer and the Pou5f1 + 40 kb enhancer, are indicated on the contact maps as bars. C and D) Comparison of the averages and SDs (size of the bars) of the distributions of physical distances between the Pou5f1 (C) and Sox2 (D) promoters (0 kb) with nearby genomic locations obtained from single molecule experiments (50) and from metainference MD based on the Micro-C maps. E and F) Scaling of contact frequency (E) and average end-to-end distance <*R*> from metainference MD as a function of genomic distance (Pou5f1, solid line; Sox2, dashed line). Dotted lines correspond to contact frequency ∼1/*L* and <*R*> ∼ *L*^1/3^. G) Representative trajectories and snapshots of the Sox2 (left) and Pou5f1 (center) genomic loci, and the corresponding EP distance distributions (right) computed from the metainference MD ensembles. In the simulation snapshots, we highlighted the promoters, the Sox2 enhancer at +100 kb, and the Pou5f1 enhancer at +40 kb (color scheme as in panels A and B).

We next applied Hi-C metainference to explore the 3D organization of the 300 kb Pou5f1 and Sox2 genomic loci of mESCs by integrating the corresponding Micro-C data (46) into our 1 kb chromatin model. Together with Nanog, discussed next, the Pou5f1 and Sox2 genes express the core TFs maintaining pluripotency in embryonic stem cells (54). Their active state is maintained by distant enhancer elements (50, 55), making these ideal systems to investigate the function of chromatin spatial organization. Metainference using 128 replicas produces contact maps in good agreement with the experiments (Fig. 3A and B). In Fig. 3C and D, we compared the distributions of the physical distances between the promoters and nearby genomic locations with single cell measurements made when the genes are actively expressed (50), finding that both averages and SDs are in good agreement with these experiments. These results suggest that the transcription status of the genes has a minor impact on the experimentally measured EP distances at these loci, consistent with recent data (56, 57).

Except for very small genomic distances *L*, we find that the studied genomic loci are spatially organized as a crumpled globule (47), with the frequency of contacts scaling as ∼1/*L* (Fig. 3E, as over very large distances (15)) and the average end-to-end distances scaling as <*R*> ∼ *L*^1/3^ (Fig. 3F). Such scaling was also found in a recent in vivo microscopy study of a *Drosophila* gene (22), highlighting how genes adopt relatively compact conformations favoring the formation of direct contacts between distant CREs. Despite such compact scaling, chromatin explores very heterogeneous conformational ensembles, sampling not only globular domains characterized by extensive contacts between distant regions, but also more open conformations with fewer contacts (see snapshots in Fig. 3G). As indicated by the rapid decay of the EP distance autocorrelation functions (Fig. S3A), these loci typically undergo fast transitions between conformations with small (<50 nm) and large (>300 nm) EP distances (Fig. 3G), over a timescale of less than a second, much faster than the typical timescales of gene bursting dynamics (58). Interestingly, the distributions of EP distances peak around ∼150 nm (Fig. 3G, left), close to the size of the condensed but liquid domains experimentally observed in active chromatin (59). This suggests an active role of such liquid domains in CRE communication.

**Fig. 4.**
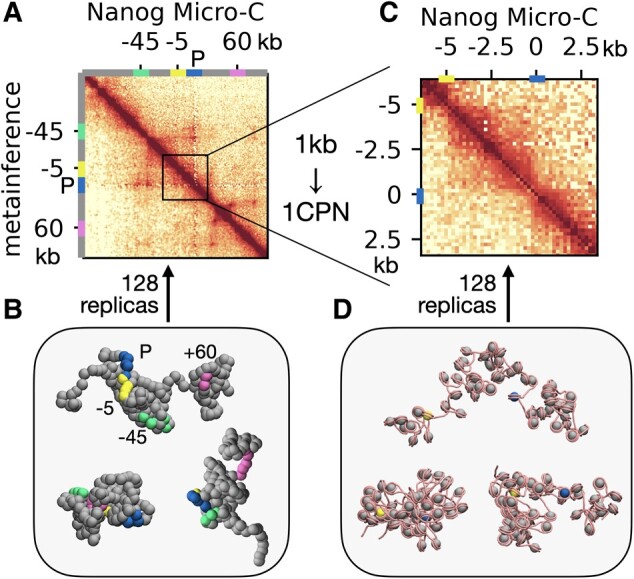
Multiscale Nanog locus organization. A) Comparison of the 200 kb Nanog locus Micro-C map as observed in mESCs with the result form metainference using *N* = 128 replicas and the 1 kb model. The locations of the Nanog promoter and the −45, −5, and +60 kb enhancers are indicated on the contact map axes with bars. B) Snapshots of representative Nanog conformations. CRE locations are highlighted with the same color scheme as in A. C) Comparison of Micro-C and 128-replica metainference contact maps applying the 1CPN chromatin model to the 10 kb locus including the Nanog promoter and its closest enhancer 5 kb upstream. D) Representative 1CPN conformations of the 10 kb locus sampled by metainference.

We then applied Hi-C metainference to study the structural ensemble and the kinetics of the 200 kb Nanog locus of mESCs. Nanog gene expression is regulated by three enhancers, located at −45 kb (E1), −5 kb (E2), and +60 kb (E3) relative to the promoter (55). This locus represents a model system to explore the coupling between 3D gene organization and function, and in particular, the potential cooperativity of the three enhancers. Figure 4A shows the good agreement between locus contact frequencies from Micro-C (46) and from the metainference simulations obtained using *N* = 128 replicas. As in the cases of the Sox2 and Pou5f1 loci, the Nanog locus is characterized by a heterogeneous conformational ensemble, with dynamic domains bounded at variable locations (Fig. 4B). Reflecting this heterogeneity, the enhancers sample a wide variety of distances to the promoter (Fig. S4A), sometimes even <50 nm, indicating the possibility of direct physical contacts. Notably, all CREs often cluster together into a single hub (Fig. 4B, bottom left snapshot, and Fig. S4B for the free energy landscape projected on a pair of EP distances), suggesting that the three enhancers may cooperatively activate their target (60), or compensate for each other's loss (55). As the other loci, the dynamics of the Nanog gene is fast, with the system able to explore highly diverse configurations on the second timescale (distance autocorrelations in Fig. S4C).

To gain insights into the finer organization of the Nanog locus at nucleosome resolution, we ran further 1CPN (35) metainference simulations of the 10 kb region encompassing the promoter and its closest enhancer located 5 kb upstream (−5 kb), which is also the most critical one for Nanog expression (55). As in the 1 kb model, we find a good agreement between the metainference and reference Micro-C map at 200 bp resolution (Fig. 4C), while the ensemble of chromatin conformations (further discussed in the next section) is also significantly heterogeneous (Fig. 4D).

### Nanog organization at nucleosome resolution reveals functional chromatin condensation at local and global scales

**Fig. 5.**
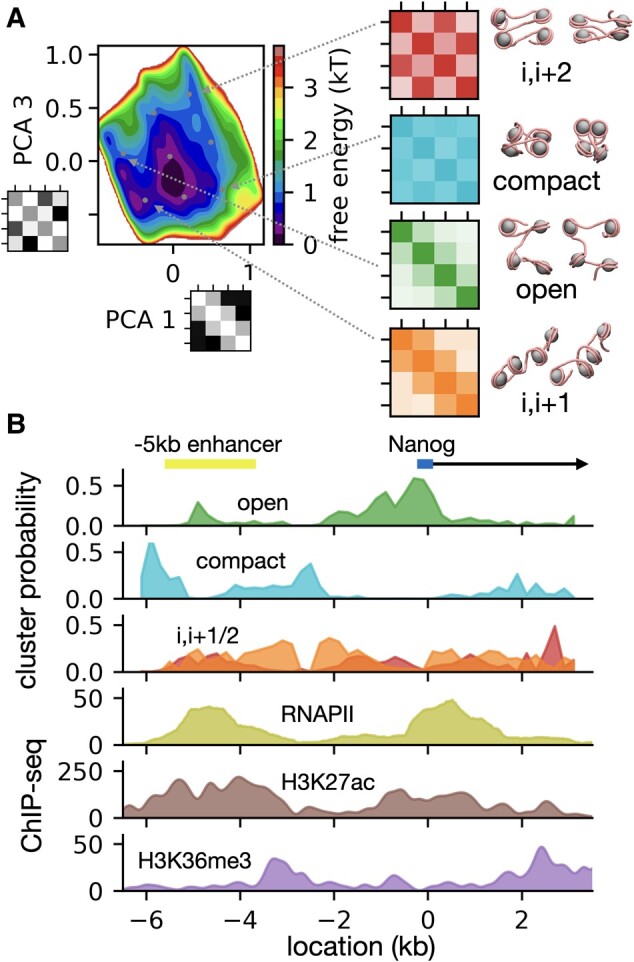
Nanog local chromatin organization. A) PCA (51) of the 10 kb Nanog locus tetranucleosome structures based on their contact maps. The left panel shows the free energy landscape along the first and third PCA components (the maps next to the labels indicate the component eigenvectors). PCA 1 captures the total number of contacts, while PCA 3 correlates with the difference between *i*, *i* + 2, and other (*i*, *i* + 1 or *i*, *i* + 3) contacts. The landscape shows eight *k*-means clusters (51) (cluster centers as circles) stabilized by different nucleosome–nucleosome interactions. On the left, we highlight contact maps and representative structures of four tetranucleosome clusters, characterized by regular *i*, *i* + 2 contacts, compact structure, open structure, or regular *i*, *i* + 1 contacts. B) Top three panels: probabilities of the four tetranucleosome clusters highlighted in (A) as a function of genomic location within the 10 kb Nanog locus. Three bottom panels: the experimental ChIP-seq signals of RNAPII binding (GEO accession: GSE22562 (61)), and H3K27ac (GEO accession: GSM2417096 (62)) and H3K36me3 (GEO accession: GSM2417108 (62)) epigenetic modifications. On top of the graphs, we also indicate the locations of the Nanog promoter and the −5 kb enhancer region (63).

From the 1CPN metainference simulations, we first explored the local organization of the Nanog locus chromatin fibers. We focused our analysis on tetranucleosomes as the fundamental units of the fiber organization (9–11, 13). We performed principal component analysis (PCA) (51) of all tetranucleosome contact maps within the 10 kb Nanog locus in all the 128 metainference replicas. The free energy landscape (logarithm of probability distribution) along the tetranucleosome projections on PCA components 1 and 3 highlights distinct populations of structures stabilized by different internucleosome contacts (Fig. 5A, left panel, eight clusters identified by *k*-means clustering (51) based on their contact maps, four of them considered for further analysis). PCA 1 represents roughly the overall number of internucleosome interactions, while PCA 3 represents the difference between *i*, *i* + 2 contacts, and other (*i*, *i* + 1 and *i*, *i* + 3) contacts (PCA 2 is not symmetric under a change in DNA direction, so it has been excluded from the analysis, Fig. S5). Notably, the landscape includes significant populations of tetranucleosomes resembling experimentally determined structures stabilized by either *i*, *i* + 1 (9) (Fig. 5A, bottom right), or *i*, *i* + 2 (10, 11) (Fig. 5A, top right) stacking interactions, which if propagated would lead to the formation of regular 1-start and 2-start chromatin fibers (64), respectively. However, the most populated structures in the landscape are actually stabilized by a mixture of *i*, *i* + 1 and *i*, *i* + 2 interactions, indicative of an overall disordered chromatin fiber organization (as can also be appreciated from the representative Nanog locus conformations in Fig. 4D). Interestingly, we also find a population of compact tetranucleosomes with all-to-all direct interactions, and another population of very open tetranucleosomes completely lacking internucleosome contacts (Fig. 5A, two middle panels on the right). As suggested based on Hi-CO experiments on yeast (13), local fiber organization correlates with chromatin epigenetic features. We find that the open tetranucleosomes are prevalently observed at the CREs of the locus, as determined from the ChIP-seq profiles of RNAPII binding (61) and H3K27ac tail modification (62) (Fig. 5B). Such open chromatin at CREs may facilitate TF and RNAPII binding, which in turn could lead to the formation of nucleosome free regions or fragile nucleosomes further disrupting the fiber (65). On the contrary, compact tetranucleosomes are prevalently found outside CREs (Fig. 5B). Such compact structures may facilitate the communication between CREs. Analysis of the average distances between any given nucleosome and the nearest one in space confirms that chromatin is more open at CREs and more compact in between (Fig. S5B). The average nearest nucleosome distance throughout the locus is ∼12 nm, consistent with the value obtained in our prior 1CPN simulations and the experimental value obtained by in situ cryo-ET of human T-lymphoblast interphase chromatin (12).

**Fig. 6.**
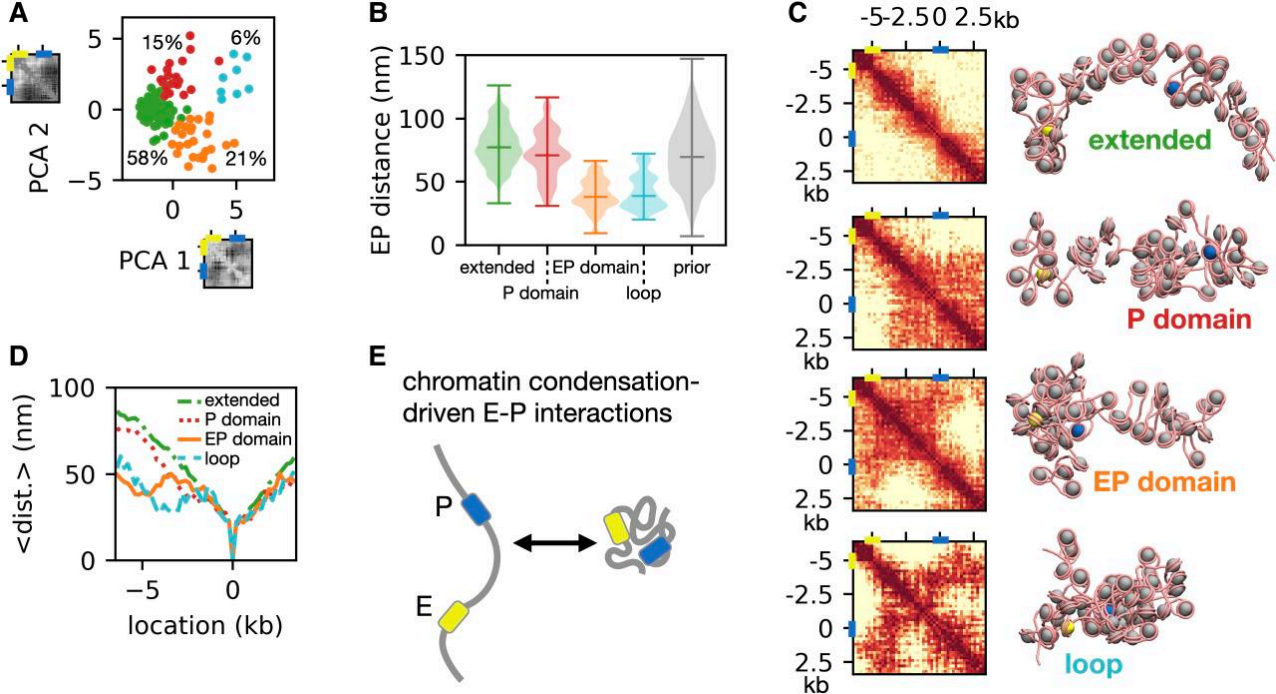
Global organization of the 10 kb Nanog locus at nucleosome resolution. A) Projection of the 10 kb Nanog locus chromatin conformations along the first two PCA coordinates obtained from their global contact maps. The component eigenvectors are shown next to the respective labels. The projection reveals four *k*-means clusters characterized by different global 3D organization (the clusters are indicated by different colors, together with their population). B) Distributions of EP distances across the four clusters highlighted in (A), together with the overall distribution for the prior 1CPN simulations. The bars in the violin plots indicate the medians and the extrema of the distributions. The four clusters are referred to as extended, promoter (P) domain, EP domain, and loop. C) Population-averaged contact maps and representative structures of the four clusters. The nucleosomes corresponding to the promoter and enhancer locations are highlighted. D) Average contact frequencies and average distances to the promoter for the four clusters. E) Schematics highlighting that direct EP contacts are driven by the condensation of the chromatin structure into a compact globule.

How does chromatin structure support the formation of direct contacts between promoter and −5 kb enhancer? To discuss this question, we performed PCA dimensionality reduction of the complete 10 kb chromatin contact maps observed in the 1CPN metainference simulations (Fig. 6A). *K*-means clustering using the top four PCA components and visual inspection of the PCA projection highlights four clusters characterized by distinct EP distances (Fig. 6B) and chromatin organization (Fig. 6C). The respective cluster-averaged contact maps and representative conformations (Fig. 6C) provide insights into the organization: in the extended cluster (58% probability), nucleosomes mainly interact with their closest neighbors and chromatin is relatively open; in the promoter (P) domain cluster (15%), there are some contacts between the promoter and the gene body downstream from it; the EP domain cluster (21%) is characterized by a distinct compact domain bounded by the −5 kb enhancer and the Nanog promoter; the loop cluster (6%) has a hairpin-like organization where chromatin adopts a condensed configuration with extensive internucleosome interactions between distant regions. In the extended and P domain clusters, the physical distances between promoter and enhancers are typically between 50 and 100 nm, while in the EP domain and loop clusters, they are <50 nm (Fig. 6B), sufficient for direct contacts mediated by the RNAPII–mediator complex (66). We note how the global organization of EP domain mirrors the folding of tetranucleosomes at the lower scale: with open chromatin motifs at the CREs establishing the boundaries of a central domain rich in compact tetranucleosomes. The condensed nature of EP domain and loop conformations is well illustrated by the fact that the average distances from the promoter do not significantly depend on genomic distance (Fig. 6D). These data reveal the high plasticity of chromatin within the Nanog locus, with predominant extended conformations that transiently condense into compact domains supporting direct contacts between enhancer and promoter (Fig. 6E). While the timescale of our metainference simulation is not sufficient to observe dynamic exchanges between these four clusters in the same trajectory, the absence of high barriers in the free energy landscape (Fig. S6A) suggests that such transitions should be diffusion limited and relatively rapid. Notably, reversible chromatin condensation is indeed observed in our unbiased 1CPN simulations, where starting from extended conformations, the system transiently collapses into compact domains on a submillisecond timescale (Fig. S6B). Furthermore, the probability distributions of EP distances (separated by 5 kb) in the metainference and in the unbiased 1CPN simulations (Figs. 6B and S6C) are very similar (*P* > 0.05). This indicates that the intrinsic molecular features of chromatin fibers, namely nucleosome plasticity and nucleosome–nucleosome interactions, are sufficient to explain the overall organization of the 10 kb Nanog locus into a mixture of extended and condensed configurations.

## Discussion

In this study, we demonstrated the successful application of metainference to integrate Hi-C contact frequencies into MD simulations of chromatin. Within this Bayesian scheme, the original polymer model of chromatin constitutes our prior, while the polymer conformations are sampled from the posterior probability distribution accounting for the contact frequencies observed in the experimental Hi-C map. This makes our approach both bottom-up, starting from a physics-based prior suitable for exploring molecular mechanisms, and top-down, providing a picture of 3D genomic organization consistent with experimental data. Our tests showed that Hi-C metainference can reconstruct both the structural ensemble and the dynamics of a target system based on contact information, with accuracy increasing with the number of replicas used to model the (in principle infinite) ensemble. A related Bayesian approach to the 3D genome (43) requires comparing the results of multiple simulations using different numbers of replicas to find the optimal minimal ensemble representing the target system. Instead, metainference is valid for any number of replicas *N* > 1, although in practice we recommend setting this to the inverse of the minimum contact probability that one aims at capturing in the final 3D ensemble (*N* = 128 proved suitable for our applications). Compared with the maximum entropy principle (25), metainference does not require iterative optimization and can consistently account for both random and systematic errors within a Bayesian framework, although the two approaches are expected to give identical results in the limit of low errors and strong bias (44, 67, 68). We note that while here we solely focused on applications to genome organization, the generality of this approach makes it applicable to other situations where we may want to integrate contact information into a prior molecular model. For instance, protein cross-linking data may be used to reconstruct the structure of large molecular complexes (69).

Metainference is particularly convenient for its flexibility, in that one can integrate Hi-C data into any chromatin model suitable for the target application. As previously shown (44), an accurate prior molecular model can help getting closer to the true conformational ensemble of the system, and therefore, producing realistic representations of the genome likely benefits from the use of physics-based models rigorously parametrized based on the interactions between the fundamental components of chromatin. By adding an extra bias on top of an existing prior model, metainference can effectively account for in vivo factors that are not directly represented, such as the binding of TFs (31, 41). However, being an approach based on thermodynamic equilibrium, the kinetics of nonequilibrium processes such as cohesin loop extrusion (30) could be potentially affected. Alternatively, the differences in the conformational ensembles obtained using the physics-based models with and without metainference can guide the modeling of necessary additional factors into the prior model or suggest hypotheses on the underlying mechanism. For the applications to the genomic organization of mESCs presented here, we integrated Micro-C data (46) into two physics-based models of chromatin, the nucleosome-resolution 1CPN model (35) and a newly parametrized 1 kb model, although other models may also be employed (34, 70).

The interplay between the 3D genome organization and the regulation of gene expression is a fundamental problem in molecular biology, for which a clear mechanistic understanding remains elusive. As observed from super-resolution chromatin tracing experiments (48) and other polymer modeling approaches (31, 43), we confirm that functional genomic interactions occur within the context of a highly heterogeneous conformational landscape that includes both open and compact conformations. In the studied loci, we find frequent EP contacts separated by a short distance on the order of the mediator–RNA polymerase complex (∼50 nm) (66), indicating their potential to directly control gene expression based on proposed models of enhancer action (71). At the Nanog locus, the chromatin often collapses into a compact structure where all three enhancers are at close distance to the Nanog promoter, possibly representing a cooperative transcription hub (60). Overall, the studied loci are organized as crumpled globules (47), with hierarchically formed globular domains encompassing increasing genomic lengths. Such fractal-like organization was already suggested based on Hi-C data (15), and further confirmed by a recent microscopy study in *Drosophila* (22), where it was shown to promote the communication of distant CREs.

Past research offers contradictory evidence (72, 73) on the importance of 3D genome structure for gene regulation, with various mechanistic models of enhancer activity still being currently debated (74). Issues may be partially due to the nonlinearity of EP communication (21, 75), with contact frequencies affecting gene expression levels only within a narrow range of values. This nonlinearity has been suggested to originate from a separation of timescales between genomic structural fluctuations and gene activation (21). Indeed, consistently with recent simulations (70), we find that the Sox2, Pou5f1, and Nanog loci of mESCs can rapidly explore their vast space of 3D conformations. Transient contacts between CREs (potentially stimulating gene expression) are formed and broken on a timescale of less than a second, significantly faster than the timescales of gene activation, on the order of several minutes (58). This separation of timescales helps explain difficulties in the experimental detection of correlations between 3D genome structure and gene expression (56, 57).

Our 1CPN simulations of the Nanog locus offer an opportunity to explore how the detailed molecular structure of chromatin can support the functional organization of genes across multiple length scales. We find that the extent of internucleosome interactions critically shapes both the local and the global folding of the chromatin fiber, leading to a surprising conformational plasticity. At the local level, chromatin often samples tetranucleosome motifs stabilized by local nucleosome–nucleosome interactions at well-defined orientations, resembling those characterized in X-ray and Cryo-EM studies (9–11), while the heterogeneous chromatin ensemble beyond the local organization is essentially disordered. This reconciles the picture of ordered chromatin from in high-resolution in vitro studies with the observations from experiments in situ, which reveal a disordered (12), liquid-like organization (59). Importantly, we find that open chromatin motifs lacking internucleosome contacts are predominantly observed at CREs. Here, specific epigenetic modifications, such as histone tail acetylation, promote the opening of chromatin (76, 77), which in turn should facilitate the recruitment of TFs and RNA polymerases and contribute to further opening (65). On the other hand, compact tetranucleosome motifs are enriched in between CREs, where they may help drive these elements into closer spatial proximity. Such kind of epigenetic-dependent local chromatin organization was previously suggested in yeast based on simulations and Hi-CO experimental contact frequencies (13). However, the tetranucleosome landscape observed at the Nanog locus appears richer than the binary set of alpha and beta motifs in yeast, perhaps thanks to the longer DNA linker length in mammalian chromatin (78). We note that our metainference simulations were performed using a uniform prior model lacking any representation of epigenetic modifications and TF binding that could be physically responsible for differences in chromatin compaction, indicating that the bias employed within our Bayesian framework can be interpreted as an effective representation of forces acting differentially along the genome. Overall, this confirms the successful integration of experimental contact frequencies to account for key factors missing in the model to produce a conformational ensemble close to that of actual in vivo conditions.

At the global level, cohesin loop extrusion (30, 79) and bridging interactions mediated by proteins (31, 80) have been previously recognized as key drivers of long-distance communication. However, our 10 kb Nanog locus simulations suggest that communication over these relatively short distances relies on dynamic transitions between extended chromatin fibers and condensed domains characterized by direct EP contacts. Interestingly, such plasticity is also observed in 1CPN simulations of chromatin lacking any experimental bias (Figs. 6B, S6B and C). Based on this similarity, we suggest that internucleosome interactions alone are sufficient to explain the observed dynamic condensation of the 10 kb Nanog locus. This is consistent with the intrinsic propensity of nucleosomes to condense into droplets by liquid–liquid phase separation (49, 81), suggested to be a key driving force of chromatin organization both at the global level of entire chromosomes (18, 25) and at the local level of individual genes (16). The detailed cell type–dependent organization of chromatin will then be shaped by epigenetic modifications of individual nucleosomes (17, 49), nucleosome positioning (82, 83) and TF binding (46), features that are implicitly accounted for by our metainference bias. Fitting into this, our simulations reveal a hierarchical organization with a tight coupling between the local and global levels: in the 10 kb Nanog locus, open tetranucleosome motifs at CREs, together with compact motifs in between, orchestrate the formation a condensed domain bringing together enhancer and promoter.

## Conclusion

We showed how Hi-C metainference allows the efficient integration of experimental data and physical modeling to explore the mechanisms underlying the multiscale spatial organization of the genome. Our simulations reveal a condensed, liquid-like chromatin whose structure and dynamics are ideally suited to support genomic function, in line with the picture recently emerging from experiments (59, 81, 84). Ultimately, our integration of theoretical modeling and experimental data will aid our understanding of the physical basis of gene regulation (71) and other key genomic functions.

## Materials and methods

### Hi-C metainference theory

In Hi-C metainference, we sample the target system by MD simulation according to Bayesian posterior probability distribution (see SI) of the conformations given the observed experimental map of frequencies *f_ij_* (1 ≤ *i*, *j* ≤ *L*). To do this, the potential energy of *N* replicas is defined from the negative logarithm of the posterior as (44):


(1) $$E(X,\;\sigma ,\;\alpha )=-\overset{N}{\underset{r=1}{\sum }}\;\log\;P(X_{r},\;\sigma_{r},\;\alpha )+\overset{L}{\underset{i=1}{\sum }}\;\overset{L}{\underset{\;j<i}{\sum }}\;[\;f_{ij}-\alpha {\bar{c}}_{ij}(X){]}^{2}\overset{N}{\underset{r=1}{\sum }}\frac{1}{2\sigma_{ijr}^{2}}$$

where the first term constitutes our prior, including the interaction potential of the original chromatin model, $U(X_{r})=-\log\;P(X_{r})$, and the negative logarithm of the hyperparameter priors; *f_ij_* and ${\bar{c}}_{ij}$ are, respectively, the experimental contact frequencies and the simulation contact probabilities between genomic locations *i* and *j* (the latter is our forward model); $\sigma_{ijr}^{2}\;=\;(\sigma_{ijr}^{B}{)}^{2}\;+\;(\sigma_{ij}^{\mathrm{SEM}}{)}^{2}$ represents the total error on the *i*–*j* contact from replica *r*, resulting from the combination of systematic error (*B*) and SEM.

For the forward model, we assume that the contact frequency (expressed as the number of sequencing reads) is estimated to be on average $\alpha \bar{c}(X)$, with α a proportionality constant to be determined, and the contact probability $\bar{c}$ computed from an average over the replicas of the kernel function:


(2) $$c(d(X))=\frac{1}{1+{\left((d-d_{0})/r_{0}\right)}^{6}}$$

This function is close to 1 and 0 for short and large distances *d* between genomic loci, respectively, with *d*_0_ representing the distance at which the kernel is equal to 1, and *r*_0_ controlling its slope. Although these parameters depend on the experimental procedure and are in principle unknown, it is reasonable to assume that they should be close to the spatial resolution of the experiment, set by the typical distances over which cross-linking may occur. This kernel has been traditionally used to setup collective variables for enhanced sampling all-atom MD simulations (85).

During the simulation, the SEMs are estimated directly from the conformational ensemble, while systematic errors are sampled according to the posterior distribution using a noninformative prior via a Monte Carlo scheme. The scaling factor *α* is also sampled by Monte Carlo each step using a uniform prior. We implemented the Hi-C metainference protocol in PLUMED 2 (85) based on the existing metainference routines.

### MD simulation details

In the 1CPN model (35), the nucleosome core particle is represented as a single ellipsoidal rigid body with orientation-dependent interactions with other nucleosomes, while linker DNA as a twistable chain where each bead represents 3 bp. Importantly, the 1CPN model can account for spontaneous DNA sequence dependent nucleosome sliding (6), effectively allowing changes in linker DNA length. In the 1 kb model, chromatin is represented as a beads-on-a-string polymer model with interactions parametrized via a bottom-up procedure from 1CPN simulations of 50 nucleosome fibers (Fig. S1A–C) with a nucleosome repeat length of 200 bp, close to the average value observed in experiments of mESCs (78). The 1 kb beads (each representing of 5 nucleosomes plus linker DNA) have a size of *a* = 22 nm, and interact via bond and angle potentials whose parameters are optimized to reproduce the corresponding distance and angle distributions from the 1CPN simulations (Figs. 1C, S1B and C), and also via nonbonded Lennard–Jones interactions with strength set so that the polymer size scales as in the theta condition (size ∼*L*^½^, Fig. S1D). In the copolymer model used for Hi-C metainference validation, the beads within the three sticky CREs have enhanced nonbonded interactions among themselves, while all other parameters are as in the prior 1 kb model. All simulations have been performed using LAMMPS (86) patched with PLUMED 2 (85) under physiological conditions (300 K temperature, and, for the 1CPN model, 150 mM ionic strength), integrating the equations of motion by Langevin dynamics.

Using the prior 1 kb chromatin model, we applied Hi-C metainference to reconstruct the 3D organization of the Pou5f1 (chr 17: 35,420–35,720 kb), Sox2 (chr 3: 34,620–34,920 kb), and Nanog (chr 6: 122,600–122,800 kb) genomic loci of mESCs (mm10). We also employed 1CPN (35) metainference simulations to explore in detail the organization of a 10 kb Nanog region (chr 6: 122,701–122,711 kb) encompassing the promoter (chr 6: 122,707 kb) and its closest enhancer located 5 kb upstream. In these applications, we employed mESC Micro-C data (46) at either 1 kb or 200 bp resolution (for the 1 kb and 1CPN simulations, respectively), normalized by Juicebox (87). For the forward model used to estimate the contact frequencies from the chromatin ensemble, we set a size parameter of *r*_0_ = 13 nm and *r*_0_ = *a* = 22 nm for the 1CPN and the 1 kb simulations, respectively, with these values corresponding to roughly the size of a nucleosome and that of a 1 kb bead, a choice consistent with the resolution of the respective models and data. In both cases, we set the kernel shift parameter as *d*_0_ = *r*_0_/2. More details on the MD simulations can be found in the SI.

For comparison with real timescales, we rescaled the simulation time of the 1 kb model simulations so that the diffusion of chromatin within the Sox2 genomic locus matches the experimental estimate of 0.25 μm^2^/s (56) (Fig. S3B).

## Supplementary Material

pgae226_Supplementary_Data

## Data Availability

All input files, simulation trajectories, and analysis scripts for this study are included at the Zenodo repository (https://doi.org/10.5281/zenodo.11207120). Code to perform Hi-C metainference simulations with LAMMPS (86) and PLUMED (85) is maintained on Github at https://github.com/gbrandani/Hi-C_Metainference.

## Preprints

This manuscript was posted on a preprint: https://doi.org/10.1101/2023.12.01.569511.

## References

[1] Chacin E, et al 2023. Establishment and function of chromatin organization at replication origins. Nature. 616:836–842. 37020028 10.1038/s41586-023-05926-8

[2] Bonev B, Cavalli G. 2016. Organization and function of the 3D genome. Nat Rev Genet. 17:661–678. 27739532 10.1038/nrg.2016.112

[3] Finn EH, Misteli T. 2019. Molecular basis and biological function of variability in spatial genome organization. Science. 365:eaaw9498 . 10.1126/science.aaw9498 PMC7421438 31488662

[4] Luger K, Mäder AW, Richmond RK, Sargent DF, Richmond TJ. 1997. Crystal structure of the nucleosome core particle at 2.8 Å resolution. Nature. 389:251–260. 9305837 10.1038/38444

[5] Zhou K, Gaullier G, Luger K. 2019. Nucleosome structure and dynamics are coming of age. Nat Struct Mol Biol. 26:3–13. 30532059 10.1038/s41594-018-0166-x PMC7386248

[6] Brandani GB, Niina T, Tan C, Takada S. 2018. DNA sliding in nucleosomes via twist defect propagation revealed by molecular simulations. Nucleic Acids Res. 46:2788–2801. 29506273 10.1093/nar/gky158 PMC5887990

[7] Tan C, Takada S. 2020. Nucleosome allostery in pioneer transcription factor binding. Proc Natl Acad Sci U S A. 117:20586–20596. 32778600 10.1073/pnas.2005500117 PMC7456188

[8] Maccarthy CM, et al 2022. OCT4 interprets and enhances nucleosome flexibility. Nucleic Acids Res. 50:10311–10327. 36130732 10.1093/nar/gkac755 PMC9561370

[9] Soman A, et al 2022. Columnar structure of human telomeric chromatin. Nature. 609:1048–1055. 36104563 10.1038/s41586-022-05236-5

[10] Schalch T, Duda S, Sargent DF, Richmond TJ. 2005. X-ray structure of a tetranucleosome and its implications for the chromatin fibre. Nature. 436:138–141. 16001076 10.1038/nature03686

[11] Ekundayo B, Richmond TJ, Schalch T. 2017. Capturing structural heterogeneity in chromatin fibers. J Mol Biol. 429:3031–3042. 28893533 10.1016/j.jmb.2017.09.002

[12] Hou Z, Nightingale F, Zhu Y, MacGregor-Chatwin C, Zhang P. 2023. Structure of native chromatin fibres revealed by Cryo-ET in situ. Nat Commun. 14:6324. 37816746 10.1038/s41467-023-42072-1 PMC10564948

[13] Ohno M, et al 2019. Sub-nucleosomal genome structure reveals distinct nucleosome folding motifs. Cell. 176:520–534. 30661750 10.1016/j.cell.2018.12.014

[14] Dixon JR, et al 2012. Topological domains in mammalian genomes identified by analysis of chromatin interactions. Nature. 485:376–380. 22495300 10.1038/nature11082 PMC3356448

[15] Lieberman-Aiden E, et al 2009. Comprehensive mapping of long-range interactions reveals folding principles of the human genome. Science. 326:289–293. 19815776 10.1126/science.1181369 PMC2858594

[16] Harris HL, et al 2023. Chromatin alternates between A and B compartments at kilobase scale for subgenic organization. Nat Commun. 14:3303. 37280210 10.1038/s41467-023-38429-1 PMC10244318

[17] S. Park, et al, Electrostatic encoding of genome organization principles within single native nucleosomes. biorxiv 2023.12.08.570828 2023. 10.1101/2023.12.08.570828, preprint: not peer reviewed [accessed 20 May 2024].

[18] Fujishiro S, Sasai M. 2022. Generation of dynamic three-dimensional genome structure through phase separation of chromatin. Proc Natl Acad Sci U S A. 119:e2109838119. 10.1073/pnas.2109838119 PMC9295772 35617433

[19] Cavalheiro GR, Pollex T, Furlong EE. 2021. To loop or not to loop: what is the role of TADs in enhancer function and gene regulation? Curr Opin Genet Dev. 67:119–129. 33497970 10.1016/j.gde.2020.12.015

[20] Yokoshi M, Segawa K, Fukaya T. 2020. Visualizing the role of boundary elements in enhancer-promoter communication. Mol Cell. 78:224–235. 32109364 10.1016/j.molcel.2020.02.007

[21] Zuin J, et al 2022. Nonlinear control of transcription through enhancer-promoter interactions. Nature. 604:571–577. 35418676 10.1038/s41586-022-04570-y PMC9021019

[22] Brückner DB, Chen H, Barinov L, Zoller B, Gregor T. 2023. Stochastic motion and transcriptional dynamics of pairs of distal DNA loci on a compacted chromosome. Science. 380:1357–1362. 37384691 10.1126/science.adf5568 PMC10439308

[23] Imakaev MV, Fudenberg G, Mirny LA. 2015. Modeling chromosomes: beyond pretty pictures. FEBS Lett. 589:3031–3036. 26364723 10.1016/j.febslet.2015.09.004 PMC4722799

[24] Giorgetti L, et al 2014. Predictive polymer modeling reveals coupled fluctuations in chromosome conformation and transcription. Cell. 157:950–963. 24813616 10.1016/j.cell.2014.03.025 PMC4427251

[25] Di Pierro M, Zhang B, Aiden EL, Wolynes PG, Onuchic JN. 2016. Transferable model for chromosome architecture. Proc Natl Acad Sci U S A. 113:12168–12173. 27688758 10.1073/pnas.1613607113 PMC5087044

[26] Shinkai S, et al 2020. PHi-C: deciphering Hi-C data into polymer dynamics. NAR Genom Bioinform. 2:lqaa020. 33575580 10.1093/nargab/lqaa020 PMC7671433

[27] Shi G, Thirumalai D. 2021. From Hi-C contact map to three-dimensional organization of interphase human chromosomes. Phys Rev X. 11:011051.

[28] Sauerwald N, Zhang S, Kingsford C, Bahar I. 2017. Chromosomal dynamics predicted by an elastic network model explains genome-wide accessibility and long-range couplings. Nucleic Acids Res. 45:3663–3673. 28334818 10.1093/nar/gkx172 PMC5397156

[29] Baú D, et al 2011. The three-dimensional folding of the α-globin gene domain reveals formation of chromatin globules. Nat Struct Mol Biol. 18:107–115. 21131981 10.1038/nsmb.1936 PMC3056208

[30] Fudenberg G, et al 2016. Formation of chromosomal domains by loop extrusion. Cell Rep. 15:2038–2049. 27210764 10.1016/j.celrep.2016.04.085 PMC4889513

[31] Brackley CA, et al 2016. Predicting the three-dimensional folding of cis-regulatory regions in mammalian genomes using bioinformatic data and polymer models. Genome Biol. 17:59. 27036497 10.1186/s13059-016-0909-0 PMC4815170

[32] Jost D, Carrivain P, Cavalli G, Vaillant C. 2014. Modeling epigenome folding: formation and dynamics of topologically associated chromatin domains. Nucleic Acids Res. 42:9553–9561. 25092923 10.1093/nar/gku698 PMC4150797

[33] MacPherson Q, Beltran B, Spakowitz AJ. 2018. Bottom-up modeling of chromatin segregation due to epigenetic modifications. Proc Natl Acad Sci U S A. 115:12739–12744. 30478042 10.1073/pnas.1812268115 PMC6294944

[34] Arya G, Zhang Q, Schlick T. 2006. Flexible histone tails in a new mesoscopic oligonucleosome model. Biophys J. 91:133–150. 16603492 10.1529/biophysj.106.083006 PMC1479056

[35] Lequieu J, Córdoba A, Moller J, De Pablo JJ. 2019. 1CPN: a coarse-grained multi-scale model of chromatin. J Chem Phys. 150:215102. 31176328 10.1063/1.5092976

[36] Sridhar A, et al 2020. Emergence of chromatin hierarchical loops from protein disorder and nucleosome asymmetry. Proc Natl Acad Sci U S A. 117:7216–7224. 32165536 10.1073/pnas.1910044117 PMC7132128

[37] Brandani GB, Gopi S, Yamauchi M, Takada S. 2022. Molecular dynamics simulations for the study of chromatin biology. Curr Opin Struct Biol. 77:102485. 36274422 10.1016/j.sbi.2022.102485

[38] Portillo-Ledesma S, Li Z, Schlick T. 2023. Genome modeling: from chromatin fibers to genes. Curr Opin Struct Biol. 78:102506. 36577295 10.1016/j.sbi.2022.102506 PMC9908845

[39] Farr SE, Woods EJ, Joseph JA, Garaizar A, Collepardo-Guevara R. 2021. Nucleosome plasticity is a critical element of chromatin liquid–liquid phase separation and multivalent nucleosome interactions. Nat Commun. 12:2883. 34001913 10.1038/s41467-021-23090-3 PMC8129070

[40] Bascom GD, Myers CG, Schlick T. 2019. Mesoscale modeling reveals formation of an epigenetically driven HOXC gene hub. Proc Natl Acad Sci U S A. 116:4955–4962. 30718394 10.1073/pnas.1816424116 PMC6421463

[41] Shrinivas K, et al 2019. Enhancer features that drive formation of transcriptional condensates. Mol Cell. 75:549–561. 31398323 10.1016/j.molcel.2019.07.009 PMC6690378

[42] Hsieh T-HS, et al 2015. Mapping nucleosome resolution chromosome folding in yeast by micro-C. Cell. 162:108–119. 26119342 10.1016/j.cell.2015.05.048 PMC4509605

[43] Carstens S, Nilges M, Habeck M. 2020. Bayesian inference of chromatin structure ensembles from population-averaged contact data. Proc Natl Acad Sci U S A. 117:7824–7830. 32193349 10.1073/pnas.1910364117 PMC7148566

[44] Bonomi M, Camilloni C, Cavalli A, Vendruscolo M. 2016. Metainference: a Bayesian inference method for heterogeneous systems. Sci Adv. 2:e1501177. 26844300 10.1126/sciadv.1501177 PMC4737209

[45] Moller J, Lequieu J, De Pablo JJ. 2019. The free energy landscape of internucleosome interactions and its relation to chromatin fiber structure. ACS Cent Sci. 5:341–348. 30834322 10.1021/acscentsci.8b00836 PMC6396382

[46] Hsieh THS, et al 2020. Resolving the 3D landscape of transcription-linked mammalian chromatin folding. Mol Cell. 78:539–553. 32213323 10.1016/j.molcel.2020.03.002 PMC7703524

[47] Grosberg A, Rabin Y, Havlin S, Neer A. 1993. Crumpled globule model of the three-dimensional structure of DNA. Europhys Lett. 23:373–378.

[48] Bintu B, et al 2018. Super-resolution chromatin tracing reveals domains and cooperative interactions in single cells. Science. 362:eaau1783. 30361340 10.1126/science.aau1783 PMC6535145

[49] Gibson BA, et al 2019. Organization of chromatin by intrinsic and regulated phase separation. Cell. 179:470–484. 31543265 10.1016/j.cell.2019.08.037 PMC6778041

[50] Li J, et al 2020. Single-gene imaging links genome topology, promoter–enhancer communication and transcription control. Nat Struct Mol Biol. 27:1032–1040. 32958948 10.1038/s41594-020-0493-6 PMC7644657

[51] Bishop CM . 2006. Pattern recognition and machine learning. New York: Springer.

[52] Naritomi Y, Fuchigami S. 2011. Slow dynamics in protein fluctuations revealed by time-structure based independent component analysis: the case of domain motions. J Chem Phys. 134:065101. 21322734 10.1063/1.3554380

[53] Scherer MK, et al 2015. PyEMMA 2: a software package for estimation, validation, and analysis of Markov models. J Chem Theory Comput. 11:5525–5542. 26574340 10.1021/acs.jctc.5b00743

[54] Boyer LA, et al 2005. Core transcriptional regulatory circuitry in human embryonic stem cells. Cell. 122:947–956. 16153702 10.1016/j.cell.2005.08.020 PMC3006442

[55] Blinka S, Rao S. 2017. Nanog expression in embryonic stem cells—an ideal model system to dissect enhancer function. BioEssays. 39:1700086. 10.1002/bies.201700086 PMC5878941 28977693

[56] Bohrer CH, Larson DR. 2023. Synthetic analysis of chromatin tracing and live-cell imaging indicates pervasive spatial coupling between genes. Elife. 12:e81861. 36790144 10.7554/eLife.81861 PMC9984193

[57] H. Ohishi, et al 2023. Transcription-coupled changes in higher-order genomic structure and transcription 2 hub viscosity prolong enhancer-promoter connectivity. biorxiv 2023.11.27.568629. 10.1101/2023.11.27.568629, preprint: not peer reviewed [accessed 20 May 2024].

[58] Rodriguez J, Larson DR. 2020. Annual review of biochemistry transcription in living cells: molecular mechanisms of bursting. Annu Rev Biochem. 89:189–212. 32208766 10.1146/annurev-biochem-011520-105250

[59] Nozaki T, et al 2023. Condensed but liquid-like domain organization of active chromatin regions in living human cells. Sci Adv. 9:eadf1488. 37018405 10.1126/sciadv.adf1488 PMC10075990

[60] Zhu I, Song W, Ovcharenko I, Landsman D. 2021. A model of active transcription hubs that unifies the roles of active promoters and enhancers. Nucleic Acids Res. 49:4493–4505. 33872375 10.1093/nar/gkab235 PMC8096258

[61] Kagey MH, et al 2010. Mediator and cohesin connect gene expression and chromatin architecture. Nature. 467:430–435. 20720539 10.1038/nature09380 PMC2953795

[62] Chronis C, et al 2017. Cooperative binding of transcription factors orchestrates reprogramming. Cell. 168:442–459. 28111071 10.1016/j.cell.2016.12.016 PMC5302508

[63] Stamatoyannopoulos JA, et al 2012. An encyclopedia of mouse DNA elements (mouse ENCODE). Genome Biol. 13:418. 22889292 10.1186/gb-2012-13-8-418 PMC3491367

[64] Brouwer T, et al 2021. A critical role for linker DNA in higher-order folding of chromatin fibers. Nucleic Acids Res. 49:2537–2551. 33589918 10.1093/nar/gkab058 PMC7969035

[65] Ishii H, Kadonaga JT, Ren B. 2015. MPE-seq, a new method for the genome-wide analysis of chromatin structure. Proc Natl Acad Sci U S A. 112:E3457–E3465. 26080409 10.1073/pnas.1424804112 PMC4500276

[66] Richter WF, Nayak S, Iwasa J, Taatjes DJ. 2022. The mediator complex as a master regulator of transcription by RNA polymerase II. Nat Rev Mol Cell Biol. 23:732–749. 35725906 10.1038/s41580-022-00498-3 PMC9207880

[67] Cavalli A, Camilloni C, Vendruscolo M. 2013. Molecular dynamics simulations with replica-averaged structural restraints generate structural ensembles according to the maximum entropy principle. J Chem Phys. 138:094112. 23485282 10.1063/1.4793625

[68] Roux B, Weare J. 2013. On the statistical equivalence of restrained-ensemble simulations with the maximum entropy method. J Chem Phys. 138:084107. 23464140 10.1063/1.4792208 PMC3598863

[69] Krepel D, Cheng RR, Pierro MD, Onuchic JN. 2018. Deciphering the structure of the condensin protein complex. Proc Natl Acad Sci U S A. 115:11911–11916. 30385633 10.1073/pnas.1812770115 PMC6255159

[70] Forte G, et al 2023. Transcription modulates chromatin dynamics and locus configuration sampling. Nat Struct Mol Biol. 30:1275–1285. 37537334 10.1038/s41594-023-01059-8 PMC10497412

[71] Panigrahi A, O’Malley BW. 2021. Mechanisms of enhancer action: the known and the unknown. Genome Biol. 22:108. 33858480 10.1186/s13059-021-02322-1 PMC8051032

[72] Benabdallah NS, et al 2019. Decreased enhancer-promoter proximity accompanying enhancer activation. Mol Cell. 76:473–484.e7. 31494034 10.1016/j.molcel.2019.07.038 PMC6838673

[73] Alexander JM, et al 2019. Live-cell imaging reveals enhancer-dependent Sox2 transcription in the absence of enhancer proximity. Elife. 8:e41769. 31124784 10.7554/eLife.41769 PMC6534382

[74] Karr JP, Ferrie JJ, Tjian R, Darzacq X. 2022. The transcription factor activity gradient (TAG) model: contemplating a contact-independent mechanism for enhancer–promoter communication. Genes Dev. 36:7–16. 34969825 10.1101/gad.349160.121 PMC8763055

[75] Xiao J, Hafner A, Boettiger AN. 2021. How subtle changes in 3d structure can create large changes in transcription. Elife. 10:e64320. 34240703 10.7554/eLife.64320 PMC8352591

[76] Collepardo-Guevara R, et al 2015. Chromatin unfolding by epigenetic modifications explained by dramatic impairment of internucleosome interactions: a multiscale computational study. J Am Chem Soc. 137:10205–10215. 26192632 10.1021/jacs.5b04086 PMC6251407

[77] Zhang R, Erler J, Langowski J. 2017. Histone acetylation regulates chromatin accessibility: role of H4K16 in inter-nucleosome interaction. Biophys J. 112:450–459. 27931745 10.1016/j.bpj.2016.11.015 PMC5300776

[78] Voong LN, et al 2016. Insights into nucleosome organization in mouse embryonic stem cells through chemical mapping article insights into nucleosome organization in mouse embryonic stem cells through chemical mapping. Cell. 167:1555–1570. 27889238 10.1016/j.cell.2016.10.049 PMC5135608

[79] Gabriele M, et al 2022. Dynamics of CTCF- and cohesin-mediated chromatin looping revealed by live-cell imaging. Science. 376:476–501. 35420890 10.1126/science.abn6583 PMC9069445

[80] Mohana G, et al 2023. Chromosome-level organization of the regulatory genome in the Drosophila nervous system. Cell. 186:3826–3844. 37536338 10.1016/j.cell.2023.07.008 PMC10529364

[81] Gibson BA, et al 2023. In diverse conditions, intrinsic chromatin condensates have liquid-like material properties. Proc Natl Acad Sci U S A. 120:e2218085120. 10.1073/pnas.2218085120 PMC10161002 37094140

[82] Wiese O, Marenduzzo D, Brackley CA. 2019. Nucleosome positions alone can be used to predict domains in yeast chromosomes. Proc Natl Acad Sci U S A. 116:17307–17315. 31416914 10.1073/pnas.1817829116 PMC6717315

[83] Oberbeckmann E, Quililan K, Cramer P, Oudelaar AM. 2024. In vitro reconstitution of chromatin domains shows a role for nucleosome positioning in 3D genome organization. Nat Genet. 56:483–492. 38291333 10.1038/s41588-023-01649-8 PMC10937381

[84] Maeshima K, Iida S, Shimazoe MA, Tamura S, Ide S. 2023. Is euchromatin really open in the cell? Trends Cell Biol. 34:7–17. 37385880 10.1016/j.tcb.2023.05.007

[85] Tribello GA, Bonomi M, Branduardi D, Camilloni C, Bussi G. 2014. PLUMED 2: new feathers for an old bird. Comput Phys Commun. 185:604–613.

[86] Plimpton S . 1995. Fast parallel algorithms for short-range molecular dynamics. J Comput Phys. 117:1–19.

[87] Robinson JT, et al 2018. Juicebox.js provides a cloud-based visualization system for Hi-C data. Cell Syst. 6:256–258. 29428417 10.1016/j.cels.2018.01.001 PMC6047755

