# Supplementary material for: Multiscale Bayesian simulations reveal functional chromatin condensation of gene loci
Source: PNAS Nexus. 2024 Jun 6;3(6):pgae226. doi: 10.1093/pnasnexus/pgae226 (PMC11179106; doi:10.1093/pnasnexus/pgae226)
Supplement: pgae226_Supplementary_Data [file pgae226_supplementary_data.pdf]

Multiscale Bayesian Simulations Reveal Functional Chromatin Condensation of Gene Loci - SI

Giovanni B Brandani †, Chenyang Gu, Soundhararajan Gopi, and Shoji Takada

Department of Biophysics, Graduate School of Science, Kyoto University, Kyoto 606-8502, Japan

† brandani@biophys.kyoto-u.ac.jp

Hi-C metainference theory

Given the experimental contact frequency f that two genomic loci are physically close in an ensemble of cells, the posterior probability distribution of an ensemble of chromatin conformations $\mathbf{X}=[X_1,\ldots,X_N]$ is proportional to (1):

$$p(\boldsymbol{X},\tilde{\boldsymbol{f}},\boldsymbol{\sigma^B},\boldsymbol{\sigma^{SEM}}|f) \propto \prod_{r=1}^{N} p(f|\tilde{f}_r,\sigma_r^B)p(\tilde{f}_r|\boldsymbol{X},\sigma_r^{SEM})p(\sigma_r^B)p(\sigma_r^{SEM})p(X_r)$$

Where $\tilde{\boldsymbol{f}}=[\widetilde{f_1},,,\widetilde{f_N}]$ are the estimates of the contact frequencies made by each of the replicas r=1,…,N, determined using a forward model based on the chromatin conformations $\mathbf{X}$. $\boldsymbol{\sigma^B}$ and $\boldsymbol{\sigma^{SEM}}$ are respectively the systematic errors (from both forward model and experiments) and the standard errors of the mean due to the use of a finite number of replicas, respectively. The terms on the righthand side represent, for each replica, the probability of experimental contact frequency f given the forward model estimate $\tilde{f}_r$, the probability of the estimate given the chromatin conformations $\mathbf{X}$, the two prior probability distributions of the errors, and the prior probability of the chromatin conformation X_r. Integrating the posterior over the estimated contact frequencies $\tilde{\boldsymbol{f}}$ assuming Gaussian statistics, accounting for the entire LxL experimental contact map, and taking its negative logarithm then gives the total potential energy reported in the main text Methods.

MD simulation details

In the 1kb chromatin model, the beads are linked along the polymer chain by harmonic bonds with potential $E_b(r)=12k_BT(r/a-1)^2$ and by angle bonds with $E_a(\theta)=k_BT(\cos\theta+1)^2$ (with θ the angle formed by the central bead connecting its neighbors), with the 1kb bead size a=22nm. The dihedral angle distribution obtained from 1CPN is uniform, so no extra dihedral potential was required. For non-local interactions, the 1kb beads interact with other non-neighboring beads via the Lennard-Jones potential $E_{nb}(r)=1.2k_BT[(a/d)^{12}-(a/d)^6]$ within a distance cutoff of $d_{cut}=3a$. The ensemble of the 50-nucleosome 1CPN fibers used to parametrize our model has been produced from 32 trajectories of 4×10^9 timesteps, each equilibrated for 5×10^9 steps starting from extended conformations (see Fig. S1A-C for the

equilibration of the average radius of gyration, neighboring distances and angles). For the model parametrization, the bond and angle potential strengths were determined directly from the corresponding 1CPN distributions by Boltzmann inversion, while the non-bonded interaction strength for theta condition was determined from 10^7-steps 1kb model simulations with various strengths for polymer lengths of 50, 100, 200, 400, and 800 kb (Fig. S1D).

In the copolymer model used for Hi-C metainference validation, the three sticky CREs are those with bead ids 31-40 (E1), 71-80 (E2) and 151-160 (E3). In this copolymer, all non-neighboring beads interact via a Lennard-Jones potential $E_{nb}(r)=\varepsilon[(a/d)^{12}-(a/d)^6]$ for $d<d_{cut}=3a$, with strength $\varepsilon=3.2k_BT$ for CRE-CRE pairs and $\varepsilon=1.2k_BT$ for any other pair (as in our prior). The target copolymer ensemble was generated from a long simulation (10^8-steps) in the absence of metainference bias, from which we constructed a synthetic contact map using the kernel $c_{ij}=1/[1+((d_{ij}-d_0)/r_0)^6]$ with $r_0=a=22$nm and $d_0=0.5a$, and a scale factor of $\alpha=50$ (meaning that a contact probability of 1 will correspond to 50 Hi-C reads). The same kernel parameters are used for the Hi-C metainference simulations.

All our MD simulations were performed using the software LAMMPS (2) patched with PLUMED 2 (3) implementing Hi-C metainference. In the 1kb model simulations, we integrated the equations of motion by Langevin dynamics using a timestep $dt = 0.005\sqrt{m/k_BT}a$, where the mass of the 1kb beads m is set to the default mass unit of 1 g/mole. Within the simulations, we set the bead sizes to the default LAMMPS unit of 1Å, but this is later rescaled to a=22nm when measuring actual physical distances. In the 1CPN simulations, we integrated the equations of motion by Langevin dynamics using a timestep of 60 fs and the default 1CPN settings (4). We set the DNA sequence parameter controlling nucleosome sliding to that of telomeric polyTTAGGG sequences, representing an average genomic sequence with intermediate nucleosome affinity.

For the metainference applications to the Sox2, Pou5f1, and Nanog loci of mESCs, the target systems were studied at 1kb resolution with N=128 replicas by running 10^6-steps Hi-C metainference simulations (the first 10^5 steps of each simulation were discarded as equilibration). For the 1CPN metainference application to the 10kb Nanog locus, we chose the initial chromatin conformations among those sampled during the unbiased 1CPN trajectories, selecting for each of the 128 replicas the 10kb conformation with the smallest root mean square distance to the corresponding one observed at the last step of the 1kb metainference simulations (this ensures that the initial 1CPN ensemble is already in reasonable agreement with the target Micro-C map). The 1CPN Nanog simulation was 15×10^6 MD steps long and we discarded the first 5×10^6 steps as equilibration (based on the convergence of the scaling factor α). For all mESC applications, we ran simulations using 128 replicas.

Metainference allows a variety of error models and priors, and upon evaluation of convergence speed and accuracy, we chose to use a single Gaussian error model acting on all the entries of the target contact map. When integrating the Hi-C data into MD, we also include in the metainference bias distant loci with zero Hi-C reads (so pairs of loci where contacts are not observed in experiments), unless they correspond to a genomic location where all the entries are zero (which likely reflects issues with sequencing at this location). When using the 1kb model, the metainference biasing force originating from a given contact between two genomic locations is applied to the two corresponding 1kb beads, while for the 1CPN simulations the bias is applied to the corresponding nucleosome core particles.

Analysis of the copolymer trajectories by Markov state modeling

To analyze the conformational ensembles and the dynamics of the polymers for our Hi-C metainference validation, we first performed TICA dimensionality reduction (5) on the copolymer contact maps defined from the positions of every 5th 1kb beads using the kernel $c_{ij}(d_{ij})=1/(1+d_{ij}/r_0)$ with r_0=22 nm. The coordinates of the polymers in our Hi-C metainference simulations have been transformed using the same linear TICA transformation determined from the copolymer simulations. Fig. 2D and SI Fig. 2D show the free energy landscapes along the two slowest TICA coordinates for the Hi-C metainference (128 replicas) and copolymer MD simulations, respectively. To quantitatively compare the probability distributions of the metainference and target copolymer ensembles, we divided the ensembles into 50 clusters by k-means clustering (6) using the 6 slowest TICA coordinates of the copolymer system. The Hi-C metainference conformations have been equivalently divided into 50 clusters based on the same cluster centers determined from the copolymer clustering. Fig. 2E indicates the Kullback-Leibler between the metainference and copolymer equilibrium probability distributions of these 50 clusters. To evaluate the dynamics, we estimated, for the metainference and copolymer systems, the transitions probabilities between the clusters by Markov state modeling using a lag-time of 10^4 MD steps (7) (Fig. 2G from main text).

Supplementary figures

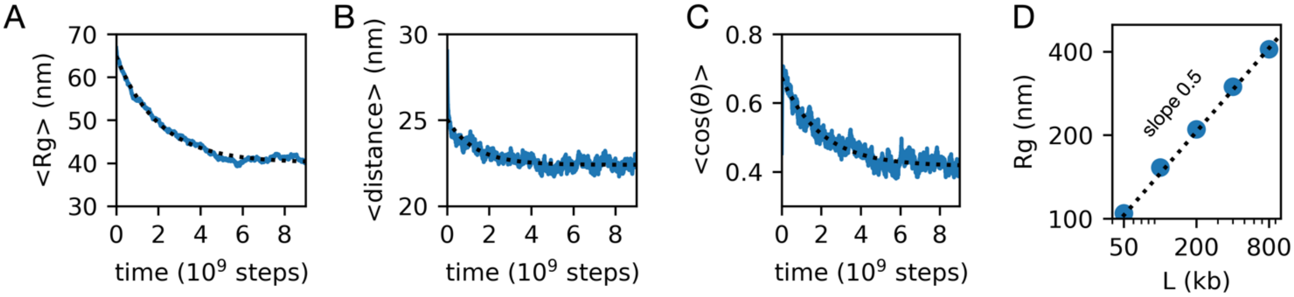

Figure S1. Unbiased chromatin fiber MD simulations. A-C. Running averages over 32 independent 50-nucleosome 1CPN trajectories showing the equilibration of radius of gyration (A), and chromatin fiber neighbor distances (B) and cosine of the angles (C) over time. To compute the distances and angles, the chromatin fiber is coarse-grained to 1kb beads, each located at the center of mass of 5 neighboring nucleosomes. The neighbor distances and angles are calculated based on the positions of these 1kb beads along the fiber (as indicated in Fig. 1A from the main text). For the parametrization of the 1kb model, the first 5×10^9 MD steps are discarded as equilibration. The dotted lines are exponential fits to the data. D. Scaling of the average radius of gyration (Rg) with chromatin fiber length in our 1kb resolution model, plotted on a logarithmic scale, based on 10^7-steps MD simulations. The slope of 0.5 indicates that the radius of gyration grows as $Rg \sim L^{0.5}$, as an ideal chain.

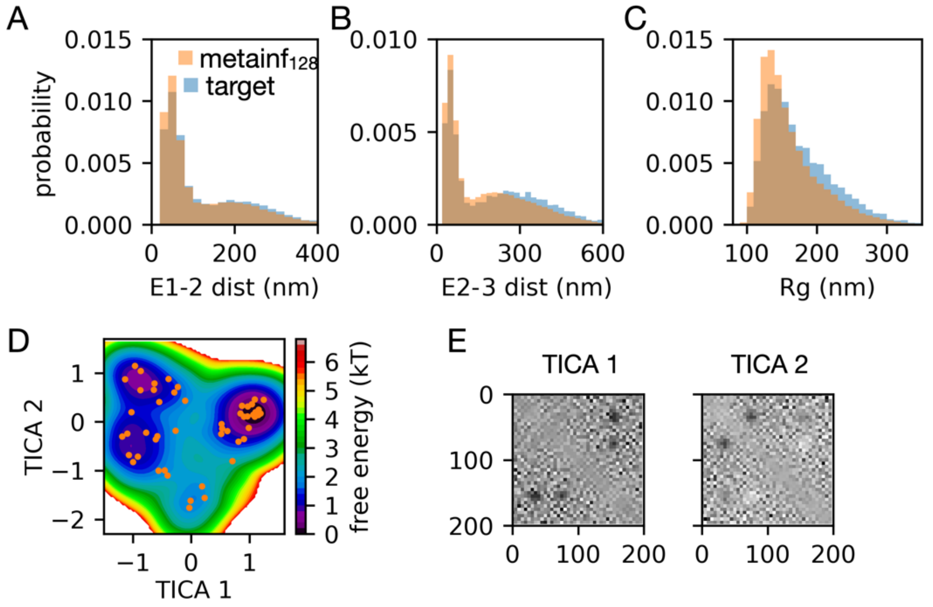

Figure S2. Hi-C metainference validation. A-C. Comparison between metainference (128 replicas) and target copolymer ensemble probability distributions of the distances between enhancer-like elements 1 and 2 (A) and 2 and 3 (B), and the radius of gyration (C). D. Free energy landscape of the target copolymer ensemble obtained by projecting the conformations along the two slowest TICA coordinates. E. Components of the two slowest TICA coordinates, capturing different interactions between the three enhancer-like elements of the copolymer.

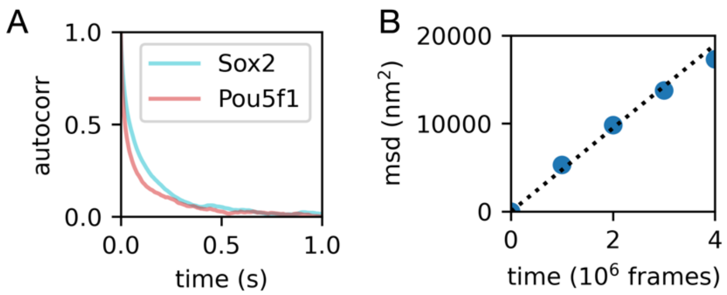

Figure S3. Hi-C metainference of the Sox2 and Pou5f1 loci of mESCs using the 1kb model. A. Time autocorrelations of the distances ($\langle \Delta d(t)\Delta d(t+\tau)\rangle_t/\langle \Delta d^2\rangle$) between the Sox2 and Pou5f1 promoters and their respective enhancers +100kb and +40kb away. B. Mean square displacement of the Sox2 enhancer-promoter 3d distance $\langle[\Delta \mathbf{r}(t+\tau)-\Delta \mathbf{r}(t)]^2\rangle_t$ as a function of simulation timestep. The scaling factor to convert simulation time to real time (reported throughout the main text) is obtained by matching the typical chromatin experimental diffusion coefficient of 0.25 $\mu m^2/s$ (8) based on a linear fit within a small time window (dotted line). TODO add comparison of Sox2 and Pou5f1 contact probability between metainference and experiments.

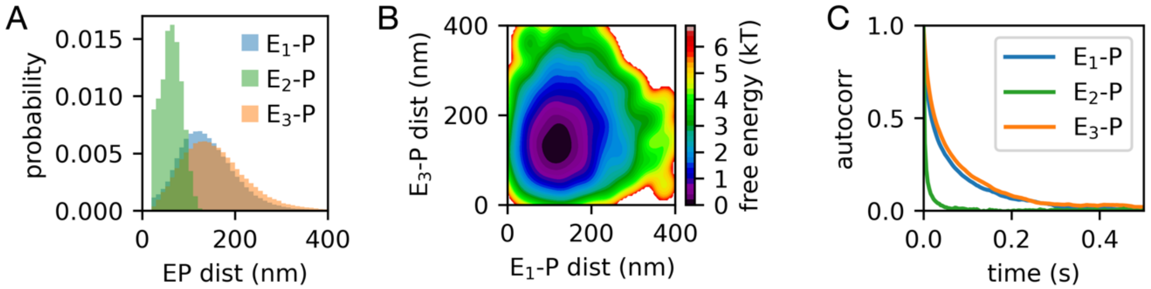

Figure S4. Hi-C metainference of the Nanog locus of mESCs using the 1kb model. A. Probability distributions of the distances between the Nanog promoter and its three enhancers located at -40kb (E_1), -5kb (E_2), and +60kb (E_3) positions. B. Free energy landscape along the distances between the Nanog promoter and the -40kb (E_1) and +60kb (E_3) enhancers. C. Time autocorrelations of the distances ($\langle \Delta d(t)\Delta d(t+\tau)\rangle_t/\langle \Delta d^2\rangle$) between the Nanog promoter and its three enhancers at -40kb (E_1), -5kb (E_2), and +60kb (E_3) positions.

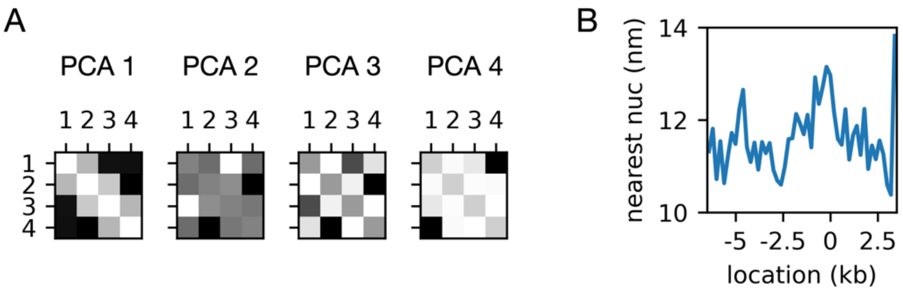

Figure S5. A. Eigenvectors of the first four tetranucleosome principal components, projected on the tetranucleosome contact maps (the coordinates used for PCA). Darker contacts correspond to higher eigenvector components. B. For each nucleosome along the 10kb Nanog locus (x-axis), the average distance to the nearest nucleosome as observed in the 1CPN metainference ensemble.

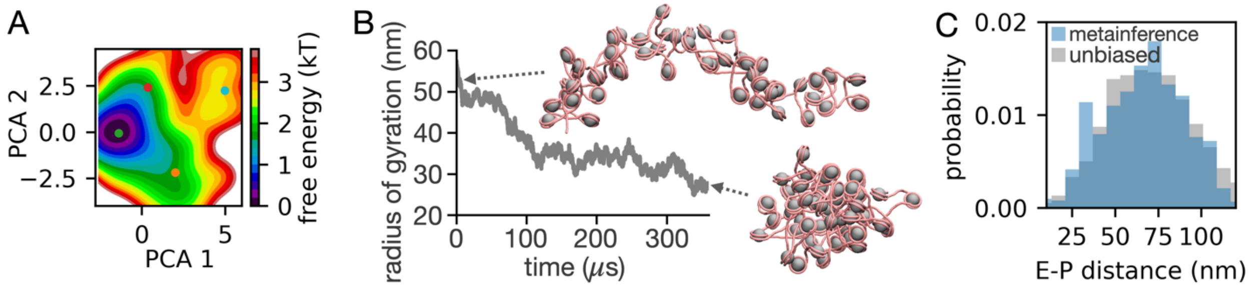

Figure S6. A. Free energy landscape of the 10kb Nanog locus from metainference simulations projected along the first two principal components obtained from the chromatin contact maps, together with the cluster centers of the 4 k-means clusters shown in Fig. 6A from the main text (using the same color scheme). B. Representative 360-μs unbiased MD trajectory using the 1CPN prior model (in the absence of metainference) and the corresponding snapshots of the initial and final chromatin configurations. C. Comparison of the probability distributions of the enhancer-promoter distances observed in the Hi-C metainference (blue) and unbiased (grey) MD simulations. The unbiased ensemble was generated from the same 36 4x10^9-steps trajectories (excluding equilibration) used to parametrize the 1kb prior model of chromatin (these are also described in SI Fig. 1). The prior 1CPN system is made of 50 nucleosomes with a uniform 200 bp nucleosome repeat region; here the enhancer-promoter distances represent the distances between the nucleosomes closest to the corresponding enhancer and promoter regions in the Hi-C metainference simulations.

References

1. M. Bonomi, C. Camilloni, A. Cavalli, M. Vendruscolo, Metainference: A Bayesian inference method for heterogeneous systems. *Sci Adv* **2**, e1501177 (2016).
2. S. Plimpton, Fast parallel algorithms for short-range molecular dynamics. *J Comput Phys* **117**, 1–19 (1995).
3. G. A. Tribello, M. Bonomi, D. Branduardi, C. Camilloni, G. Bussi, PLUMED 2: New feathers for an old bird. *Comput Phys Commun* **185**, 604–613 (2014).
4. J. Lequieu, A. Córdoba, J. Moller, J. J. De Pablo, 1CPN: A coarse-grained multi-scale model of chromatin. *Journal of Chemical Physics* **150**, 215102 (2019).
5. Y. Naritomi, S. Fuchigami, Slow dynamics in protein fluctuations revealed by time-structure based independent component analysis: The case of domain motions. *J Chem Phys* **134**, 065101 (2011).
6. C. M. Bishop, *Pattern Recognition and Machine Learning* (Springer, 2006).
7. M. K. Scherer, *et al.*, PyEMMA 2: A Software Package for Estimation, Validation, and Analysis of Markov Models. *J Chem Theory Comput* **11**, 5525–5542 (2015).
8. C. H. Bohrer, D. R. Larson, Synthetic analysis of chromatin tracing and live-cell imaging indicates perva-sive spatial coupling between genes. *Elife* **12**, e81861 (2023).
